# Age affects the contribution of ipsilateral brain regions to movement kinematics

**DOI:** 10.1002/hbm.24829

**Published:** 2019-10-16

**Authors:** Caroline Tscherpel, Lukas Hensel, Katharina Lemberg, Jana Freytag, Jochen Michely, Lukas J. Volz, Gereon R. Fink, Christian Grefkes

**Affiliations:** ^1^ Medical Faculty University of Cologne and Department of Neurology, University Hospital Cologne Cologne Germany; ^2^ Cognitive Neuroscience, Institute of Neuroscience and Medicine (INM‐3) Research Centre Jülich, Jülich Germany; ^3^ Wellcome Trust Centre for Neuroimaging, University College London London UK

**Keywords:** aging, dorsal premotor cortex, movement kinematics, online TMS interference

## Abstract

Healthy aging is accompanied by changes in brain activation patterns in the motor system. In older subjects, unilateral hand movements typically rely on increased recruitment of ipsilateral frontoparietal areas. While the two central concepts of aging‐related brain activity changes, “Hemispheric Asymmetry Reduction in Older Adults” (HAROLD), and “Posterior to Anterior Shift in Aging” (PASA), have initially been suggested in the context of cognitive tasks and were attributed to compensation, current knowledge regarding the functional significance of increased motor system activity remains scarce. We, therefore, used online interference transcranial magnetic stimulation in young and older subjects to investigate the role of key regions of the ipsilateral frontoparietal cortex, that is, (a) primary motor cortex (M1), (b) dorsal premotor cortex (dPMC), and (c) anterior intraparietal sulcus (IPS) in the control of hand movements of different motor demands. Our data suggest a change of the functional roles of ipsilateral brain areas in healthy age with a reduced relevance of ipsilateral M1 and a shift of importance toward dPMC for repetitive high‐frequency movements. These results support the notion that mechanisms conceptualized in the models of “PASA” and “HAROLD” also apply to the motor system.

## 1 INTRODUCTION

Unilateral hand movements are primarily driven by a lateralized network of cortical and subcortical areas. However, besides contralateral sensorimotor areas (Porter & Lemon, 1995), also ipsilateral brain regions contribute to the coordination of upper limb movements (Cramer, Finklestein, Schaechter, Bush, & Rosen, 1999; Tinazzi & Zanette, 1998). Bilateral recruitment patterns have particularly been observed in young healthy subjects for more complex motor tasks with higher behavioral demands (Buetefisch, Revill, Shuster, Hines, & Parsons, 2014; Catalan, Honda, Weeks, Cohen, & Hallett, 1998; Hummel, Kirsammer, & Gerloff, 2003; Verstynen, 2004).

As in more complex tasks, the enhanced brain activity of the ipsilateral motor system has frequently been encountered in association with age. Older subjects show more extensive, as well as more bilateral activation patterns (Heuninckx, 2005; Hutchinson, 2002;Mattay et al., 2002 ; Michely et al., 2018 ; Riecker et al., 2006). While at the behavioral level motor performance typically declines with increasing age (Hackel, Wolfe, Bang, & Canfield, 1992; Kolb, Forgie, Gibb, Gorny, & Rowntree, 1998; Salthouse, 2000), ipsilateral brain activity increases, comprising especially the primary motor cortex, premotor and parietal areas (Mattay et al., 2002; Michely et al., 2018; Riecker et al., 2006).

Based on neuroimaging findings which have consistently shown altered activation patterns during aging, two main models have been conceptualized: a decrease in lateralization with increasing age (“Hemispheric Asymmetry Reduction in Older Adults” (HAROLD) theory, (Cabeza et al., 1997; Cabeza, Anderson, Locantore, & McIntosh, 2002)) and a shift of activation from posterior to anterior regions (“Posterior to Anterior Shift in Aging” (PASA) model, (Davis, Dennis, Daselaar, Fleck, & Cabeza, 2008). While initially put forward in the context of cognitive tasks, these models have been readily transferred to the motor system (Heuninckx, 2005; Hutchinson, 2002; Mattay et al., 2002; Michely et al., 2018; Riecker et al., 2006; Ward & Frackowiak, 2003).

From a functional perspective, such changes observed during cognitive tasks have mostly been associated with compensatory recruitment in order to counteract aging‐related structural, functional, and metabolic changes (Cabeza et al., 2002; Grady, 2012; Reuter‐Lorenz & Cappell, 2008) with the ultimate goal of maintaining performance.

However, though intuitive, the functional significance of these age‐related activity changes associated with motor tasks remains to be established (Seidler et al., 2010). Although the principle of compensation may also be relevant for the motor system (Mattay et al., 2002), a majority of studies did not support this idea (Hutchinson, 2002; Langan et al., 2010; Riecker et al., 2006), since the authors could not link the degree of over‐activation to improved motor performance. Besides, altered brain activity may also represent dedifferentiation (Bernard & Seidler, 2012; Langan et al., 2010; Seidler et al., 2010).

Yet, the models of age‐related changes and their functional significance were primarily based on BOLD activity changes correlating with behavior. In this context, a significant limitation of functional neuroimaging studies, including analyses of functional or effective connectivity, is that they cannot derive a causal role of a particular brain region for performance. By contrast, transcranial magnetic stimulation (TMS) can be used to transiently interfere with neural activity of a brain area during task performance (Gerloff et al., 1998; Pascual‐Leone, Bartres‐Faz, & Keenan, 1999; Walsh & Cowey, 2000). By directly interfering with the fine‐tuned neural processing and thereby disturbing task performance, online TMS interference allows insights into the causal role of a specific area for a given task of interest.

We thus used neuronavigated, online TMS interference to elucidate the age‐related role of ipsilateral brain regions in healthy aging. As age‐dependent changes have been frequently reported for ipsilateral primary motor cortex (M1), dorsal premotor cortex (dPMC) and intraparietal sulcus (IPS) (Mattay et al., 2002; Riecker et al., 2006; Ward, Swayne, & Newton, 2008), we investigated their task‐related role in young and older healthy subjects while they performed three motor task of different motor demands: (a) rapid alternating pointing between two fixed targets, (b) maximum finger tapping frequency, and (c) maximum hand tapping frequency. Movement kinematics were recorded using a three‐dimensional (3D) motion analyzer system. Given the increases in frontoparietal activity reported for older subjects (Heuninckx, 2005; Mattay et al., 2002; Riecker et al., 2006), we hypothesized that TMS interference with ipsilateral frontoparietal areas would primarily disturb motor performance in older subjects, consistent with a compensatory role of activity changes in these areas. Moreover, the different motor tasks were not only designed to recruit different motor effectors ranging from the entire arm for the pointing tasks to the single index finger during the finger tapping task, but also to differentially probe the involvement of the three brain regions of interest. Accordingly, primary motor cortex was supposed to be particularly relevant for fine‐tuned repetitive finger movements which strongly rely on the modulation of interhemispheric inhibition applicable to our tapping tasks (Hinder, 2012; Liuzzi, Hörniss, Zimerman, Gerloff, & Hummel, 2011), but may be supported by premotor regions in aging. The cortex in anterior intraparietal sulcus was assumed to be particularly involved during the pointing tasks since the IPS is known to be engaged in such visuomotor tasks (Grefkes & Fink, 2005; Wang, Fink, Dafotakis, & Grefkes, 2009).

## 2 MATERIALS AND METHODS

### 2.1 Main experiment

#### 2.1.1 Subjects

Thirty‐two healthy subjects participated in this study: 15 young healthy subjects (five females, all subjects reported right‐handedness according to the Edinburgh Handedness Inventory (EHI) (laterality score ≥ 75%), mean age 27.8 ± 3.5 *SD* years; range: 24–34 years) and 17 older healthy subjects (four women, all right‐handed, mean age 63.4 ± 9.2 *SD* years; range: 51–89 years) were recruited from our subject database. Due to the procedure explained in the following, the recruitment was challenged and limited to subjects having an individual MRI scan stored in the database. Post hoc calculation of power confirmed that although the given sample size is small, based on the detected effect sizes and given an alpha‐error of 0.05, we achieved an observed power of 0.79 (G*Power 3.1). None of the subjects had a history of neurological, psychiatric, orthopedic or rheumatic disease nor any contraindication to TMS (Rossi, Hallett, Rossini, & Pascual‐Leone, 2009). All participants gave informed written consent before entering the study, which was approved by the local ethics committee (number of ethical vote: 14–141) and the experiment was carried out in accordance with the Declaration of Helsinki.

#### 2.1.2 Experimental design

We used a single‐blinded, pseudorandomized mixed design. Accordingly, the order of stimulation sites was pseudorandomized per subject before the experiment and balanced within subjects. The order of motor tasks was pseudorandomized across subjects as well. Short bursts of repetitive TMS pulses (rTMS) were administered at a frequency of 10 Hz with a stimulation intensity of 90% of resting motor threshold (see below) concurrent to task execution (Davare, Andres, Cosnard, Thonnard, & Olivier, 2006; Gerloff et al., 1998; Volz et al., 2017), starting with a visual go cue and lasting throughout the trial for 2.0–2.5 s (20–25 pulses) depending on the given motor task (see below).

Trials within a given stimulation block were separated by a 3‐s pause to minimize the likelihood of carry‐over and lasting effects (see below) (Rotenberg, Horvath, & Pascual‐Leone, 2014). The software Presentation® (Version 9.9, Neurobehavioral Systems, Berkeley, CA, http://www.neurobs.com) was used for both stimulus presentation and time‐locked triggering of the TMS machine (Volz et al., 2017).

**Figure 1:**
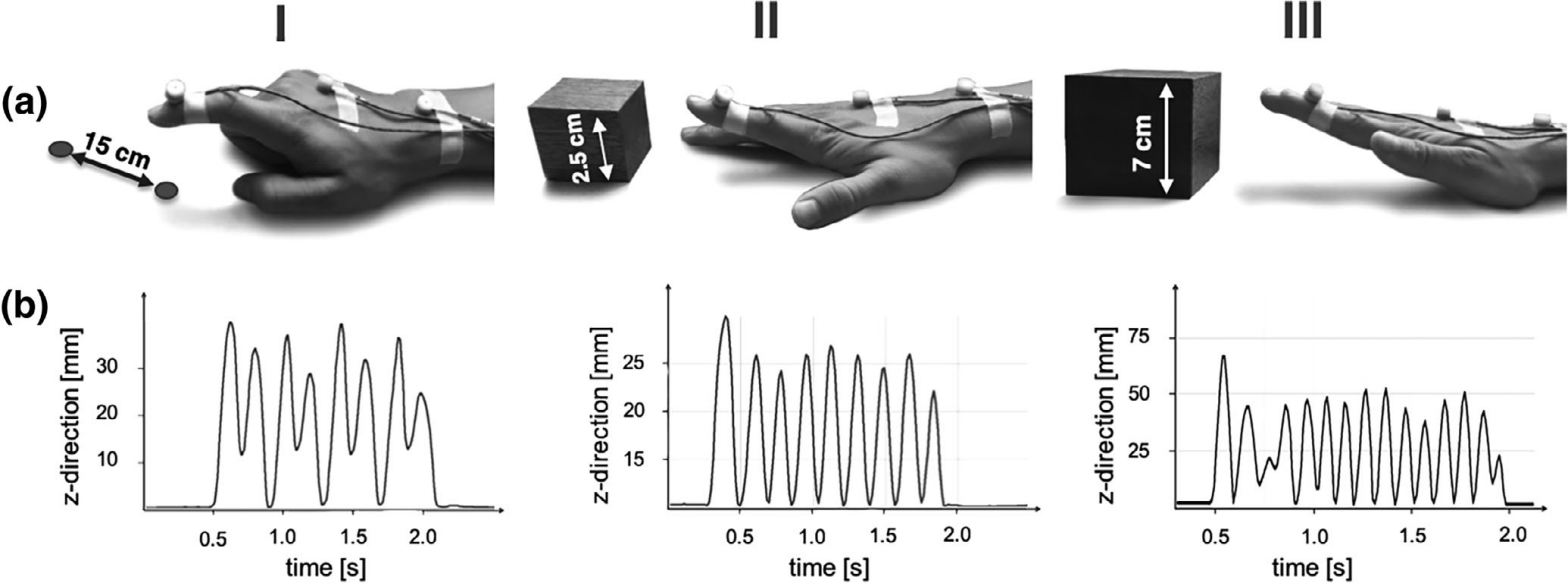
Motor task during online related TMS. (a) (I) Rapid pointing movements touching two spots with a distance of 15 cm as fast as possible. (II) Index finger tapping with a height of 2.5 cm at maximum speed. (III) Hand tapping with a height of 7 cm at maximum speed. All motor tasks were studied using a three‐dimensional motion analyzer system based on ultrasound‐emitting markers that were fixed to the dorsal side of the distal interphalangeal joint of the right index finger (marker I), the dorsal side of the third metacarpophalangeal joint (marker II), and between the styloid processes of ulna and radius (marker III). (b) Illustration of the kinematic data obtained for data analysis: traces of vertical movements (*z*‐direction) of marker I over time are shown for every motor task (I–III)

Three motor tasks of different behavioral demands were used to probe the influence of the stimulation sites on motor performance (Figure 1).

#### 2.1.3 Pointing task (I)

The rapid pointing task engaged muscle groups of the entire arm including the shoulder, and particularly required target accuracy. Therefore, it strongly relied on neural systems for action‐space representations (Colby & Goldberg, 1999; Rizzolatti, Fogassi, & Gallese, 1997), sensori‐ and visuomotor coordinate transformations, and visuospatial attention (Grefkes & Fink, 2005; Wang et al., 2009). Subjects were asked to perform repetitive sagittal pointing movements with their right index finger between two targets (Figure 1). For this purpose, we installed an apparatus with two fixed targets. The distance between the pointing targets was 15 cm in the sagittal plane and 3 cm in the vertical plane, which was visible throughout the entire block of pointing movements (Figure 1).

The difference in height ensured that participants lifted their arm, thereby avoiding sliding movements on the table. Each trial lasted 2.5 s followed by a pause of 3 s. To prevent interference with the visual target, movement onsets were indicated by a brief acoustic tone. Subjects were instructed to move as fast and as accurate as possible.

#### 2.1.4 Index finger tapping (II)

The finger tapping task tested repetitive isolated finger movements which strongly rely on the modulation of interhemispheric interactions (Hinder, 2012; Liuzzi et al., 2011). Subjects were instructed to perform repetitive index finger tapping movements as fast and accurate as possible with their right hand upon the appearance of a visual cue which was presented on a video screen controlled by the software Presentation®. Subjects placed their right palm on a defined position on the table and performed repetitive vertical movements at the metacarpophalangeal joint of their index finger (Nowak et al., 2007). A dice with a height of 2.5 cm indicated the target amplitude (Figure 1). Each tapping trial lasted 2 s. As it is assumed that effects of high‐frequency rTMS outlast the stimulation period for about half of the duration of stimulation (Rotenberg et al., 2014), tapping trials were separated with a pause of 3 s to avoid rTMS carry‐over effects and fatigue. Short finger tapping trials were used because the maximum finger tapping frequency is usually highest during the first few seconds of a trial (Wang et al., 2009).

#### 2.1.5 Hand tapping (III)

The hand tapping task was similar to the finger tapping task with respect to its cyclical, repetitive aspect. Moreover, it involved similar motor components and, therefore, additionally served as an internal control condition for effects seen in the finger tapping task. However, the hand tapping task recruited more proximal arm and wrist muscle groups, which are considered to rely somewhat less on interhemispheric interactions compared to isolated index finger movements (Aune, Ettema, & Vereijken, 2016; Harris‐Love, Perez, Chen, & Cohen, 2007; Sohn, Jung, Kaelin‐Lang, & Hallett, 2003). Subjects were asked to place their right hand on the defined position on the table and, in response to the cue, perform repetitive flexion and extension of the wrist with a stretched hand at maximum speed (Nowak et al., 2007). The target movement amplitude was indicated by a cube of 7 cm height (Figure 1). Similarly to (I), one tapping trial lasted 2 s with a pause interval of 3 s (all indicated by visual cues).

**Figure 2:**
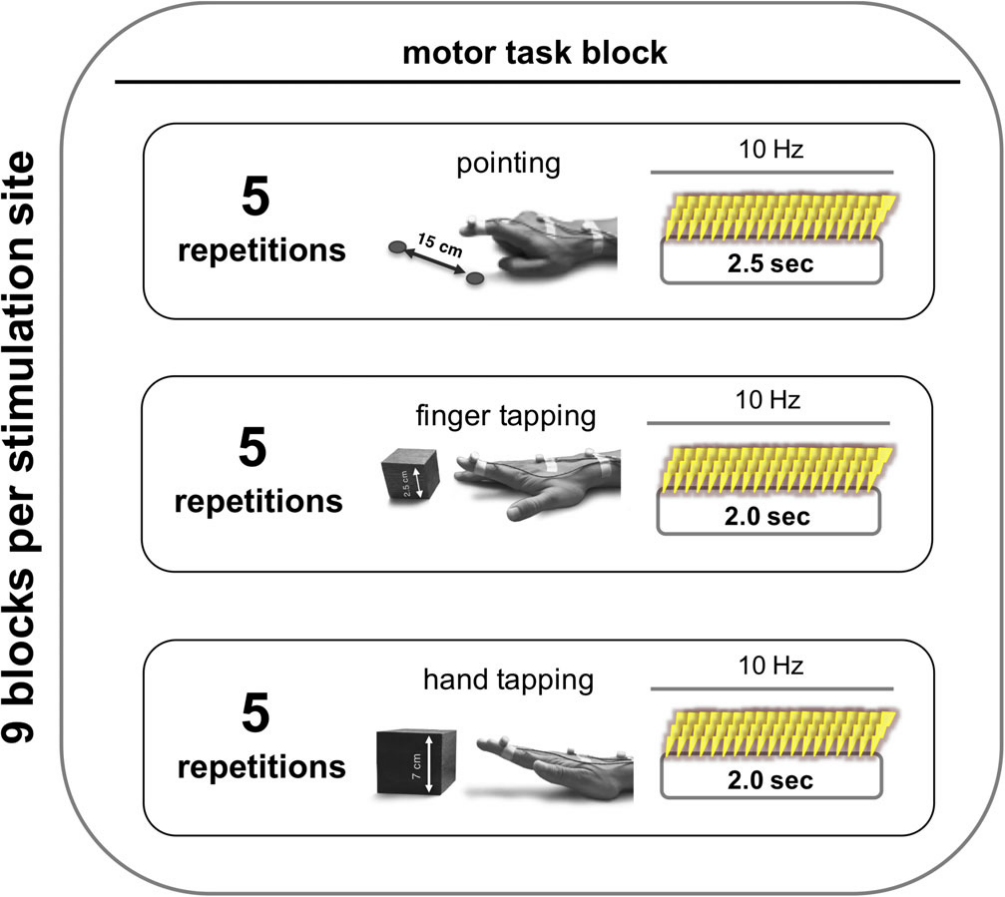
Study design. For each stimulation site, we acquired nine blocks for a given motor task. The order of stimulation sites was pseudorandomized per subject and balanced within subjects. The order of motor tasks was also pseudorandomized across subjects. One block consisted of five consecutive repetitions of one movement trial (i.e., pointing, finger tapping, hand tapping). See methods for more information

The testing battery for each stimulation site consisted of nine blocks, three repetitions of each type of movements. The order of these nine blocks per site was pseudorandomized. One block was composed of five consecutive repetitions of one movement trial (i.e., 5 times of 2.0 s of finger tapping or hand tapping, or 2.5 s of pointing) (Figure 2). This means that we acquired three blocks of five consecutively repetitions per task per stimulation site. Each trial of movement was separated by a 3‐s pause. Each block was separated by 10 s of rest. A break of 30–45 s for switching between the different stimulation sites ensured a reliable location of each region using the neuronavigation system.

Written instructions were displayed for 3 s on a video screen, indicating the upcoming block of motor task (e.g., “pointing,” “finger tapping” or “hand tapping”). Upon the appearance of the visual go cue, instructing the subject to start the requested movements, the software synchronously triggered the TMS machine to apply 10 Hz rTMS throughout the duration of a trial for 2.0–2.5 s (20–25 pulses) depending on the given motor task (see above). A visual stop signal displayed for 3 s indicated the end of a trial. Of note, for the pointing task, start and stop of the trial were also indicated by a brief acoustic tone so that the subjects could focus on the pointing apparatus.

The entire experiment lasted about 60 min (15 min per stimulation site). Before the TMS sessions, subjects were trained in all tasks until they reached a stable performance.

#### 2.1.6 3D ultrasound movement recording

Motor performance was assessed using the Zebris CMS 20 kinematic motion analyzer system (Zebris Medical Company, Isny, Germany) (Nowak et al., 2007; Wang et al., 2009). The system mainly consists of a measuring sensor, which captures with high spatial (0.1 mm) and temporal (100 Hz) resolution the 3D positions of markers, fixed on the body parts to be examined, emitting ultrasonic pulses (diameter: 5 mm, weight: 1 g) (Wang et al., 2009). For the current experiment, the 3D‐tracking markers were attached to the dorsal side of the distal interphalangeal joint of the right index finger (marker I), the dorsal side of the third metacarpophalangeal joint (marker II), and between the styloid processes of ulna and radius (marker III) (Figure 1). The *x*‐, *y*‐, and *z*‐directions of the position marker coordinates refer to the medio‐lateral, antero‐posterior, and vertical directions with regard to the subject performing the task. Kinematic data were continuously recorded throughout the entire experiment.

#### 2.1.7 Neuronavigated transcranial magnetic stimulation

TMS was performed using a Magstim Super Rapid^2^ stimulator (The Magstim Co., Ltd, Whitland, United Kingdom) equipped with a 70 mm figure‐of‐eight air film coil. Throughout the TMS sessions, the position of the coil was tracked and recorded using a frameless computerized stereotaxic neuronavigation system (BrainSight V.2.0.7; Rogue Research Ltd; Montreal, Canada). For neuronavigation, the head of the subject was coregistered with the individual high‐resolution anatomical MR image (MDEF sequence; voxel size: 1.0 × 1.0 × 1.0 mm^3^, FOV 256 mm, 176 sagittal slices, TR 2250 ms, TE 3.93 ms) via anatomical landmarks (see Nettekoven et al., 2014 for further details). The structural MRI images were available in the subject database.

The “motor hotspot” for ipsilateral M1 (i.e., right primary motor cortex) was defined as the coil position eliciting motor evoked potentials (MEP) of the highest amplitude in response to a TMS pulse applied tangentially to the skull in a 45° posterior–anterior current direction, thereby targeting the posterior wall of the precentral gyrus at the hand knob formation (Yousry et al., 1997). MEP amplitudes were assessed with biphasic pulses. In addition, MEP amplitudes of the left first interosseous (FDI) muscle were measured using Ag/AgCl surface electrodes (Tyco Healthcare) in a belly‐to‐tendon montage. The EMG signal was amplified, filtered (0.5 Hz high pass and 30–300 Hz bandpass), and digitized using a Powerlab 26T device and the LabChart software package version 8.0 (AD Instruments, Australia).

The resting motor threshold (RMT) was defined using an algorithm provided by the TMS Motor Threshold Assessment Tool (MTAT) 2.0 (http://www.clinicalresearcher.org/software.html) (Awiszus, 2003; Diekhoff et al., 2011). Using a maximum‐likelihood procedure, the algorithm proposes stimulation intensities that are subsequently tested regarding their ability to induce an EMG response higher than 50 μV, which is accordingly entered by the experimenter. The MTAT has been shown to accurately estimate motor thresholds using less stimuli than the standard 5‐out‐of‐10 rule (Awiszus, 2003).

For motor task interference, 10 Hz rTMS was applied at 90% RMT during task execution (Volz et al., 2017). A subthreshold stimulation intensity was chosen to prevent the induction of MEPs, which may irritate participants and impact on their task performance. Besides, stimulating brain regions in the hemisphere ipsilateral to the moving hand and areas beyond M1 additionally avoid the evocation of distracting muscle activity. Moreover, by using an online TMS approach, we used the immediate effects of TMS interfering with physiological neural activity underneath the stimulation coil while subjects performed a given task. The advantage of online TMS interference compared to the more widely used “offline”‐TMS approach is that by directly disturbing neural activity during task performance stimulation effects are immediately present and less prone to vary with respect to the magnitude and direction of after‐effects as observed for “offline TMS” protocols (Hamada, Murase, Hasan, Balaratnam, & Rothwell, 2013). Of note, TMS interference refers to the neurophysiological mechanisms, that is, disturbing neural activity, rather to the behavioral consequence. Besides, in contrast to the excitatory and inhibitory effects of offline rTMS (Nettekoven et al., 2015), online TMS is considered to invariably interfere with the neural processing, that is, inducing a virtual online lesion, and thereby disturb task performance (Rossi, 2004; Rossini et al., 2015; Walsh & Cowey, 2000). Importantly, there is no evidence that 10 Hz online interventions paradigms have a lasting effect on cortical excitability, which might probably be due to the fact that 10 Hz stimulation applied for increasing cortical excitability in offline‐experiments are administered in a completely different stimulation protocol (longer stimulation trains, higher number of pulses up to 1,000–2,000 in total, and often suprathreshold intensities, for a review see Fitzgerald, Fountain, & Daskalakis, 2006; Rossini et al., 2015).

Furthermore, online TMS paradigms are more flexible with regard to randomization of stimulation sites within a single session. High‐frequency rTMS trains have widely been used before in order to interfere with task performance (Davare, Andres, et al., 2006; Gerloff et al., 1998; Lotze et al., 2006; Volz et al., 2017). In contrast to single pulse TMS, 10 Hz rTMS trains increase the likelihood to effectively and temporally accurately interfere with task performance.

For online interference, the stimulator was controlled and triggered by an in‐house script using the software Presentation® to ensure reliable timing of TMS pulse applications (for further technical details see Volz et al., 2017). The stimulation was triggered by the computer synchronously with the presentation of the go cue, and was administered throughout the trial for 2.0–2.5 s (20–25 pulses) depending on the motor task (see above). Applying TMS throughout the entire duration of the motor task implicated the possibility to intervene not only with movement execution, but also preparation or sensory integration. However, it ensured an effective and comparable interference for all motor tasks, all brain regions and both groups. Of note, the in‐house script simultaneously presented the visual cues and triggered the TMS machine.

**Figure 3:**
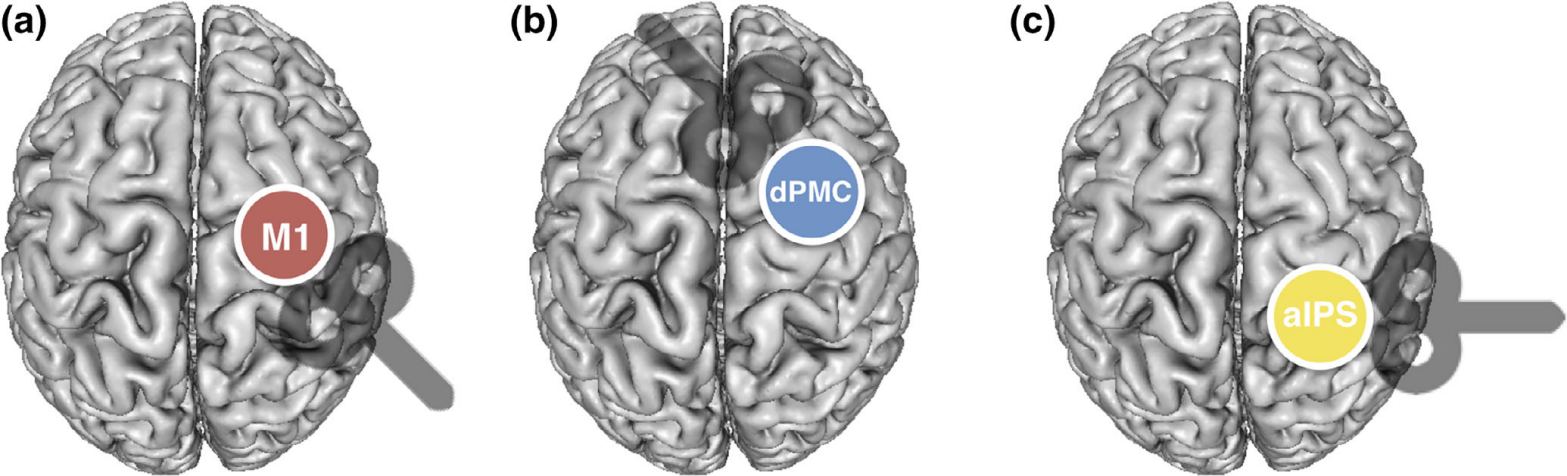
Ipsilateral stimulation sites with coil orientations. (a) Right ipsilateral primary motor cortex (M1), (b) right ipsilateral dorsal premotor cortex (dPMC), and (c) right ipsilateral anterior intraparietal sulcus (IPS)

The following stimulation sites were investigated all ipsilateral to the right (dominant) hand (Figure 3): (a) right ipsilateral M1, (b) right ipsilateral dPMC, and (c) right ipsilateral IPS. Ipsilateral M1 was equivalent to the motor hotspot as defined above.

For targeting ipsilateral dPMC and IPS, we used coordinates based on recent activation likelihood estimation meta‐analyses on hand motor fMRI activity (Hardwick, Rottschy, Miall, & Eickhoff, 2013; Rehme, Eickhoff, Rottschy, Fink, & Grefkes, 2012). For each subject, MNI (X/Y/Z) coordinates (dPMC: 38/6/62; IPS: 42/−40/50) were warped into individual 3D‐space of the anatomical MRI using Statistical Parametric Mapping (SPM12, http://www.fil.ion.ucl.ac.uk/) and MRIcron (Neva, Brown, Mang, Francisco, & Boyd, 2017).

For interference with ipsilateral dPMC activity, the coil was held tangentially to the skull in a 45° anterior–posterior position perpendicular to the course of the precentral sulcus. This position warranted optimal stimulation of dPMC neurons situated in the anterior wall of the precentral gyrus (close to the intersection with the superior frontal sulcus). By contrast, for stimulation of ipsilateral anterior IPS, the coil was held in a 90° latero‐medial position, perpendicular to the intraparietal sulcus, leading to a preferential stimulation of neurons situated in the medial IPS wall, probably equivalent to macaque area MIP which is engaged in reaching movements and visuomotor transformation (Colby, 1998; Grefkes & Fink, 2005; Grefkes, Ritzl, Zilles, & Fink, 2004). Similar to M1, the stimulation intensity for ipsilateral dPMC and IPS was applied at the same intensity, that is, 90% RMT obtained at M1 for a given subject. Using the same stimulation intensity for each site ensures that sensory stimulation effects like acoustic noise or tactile sensations remain comparable between stimulation sites within a given subject. Hence, between‐region differences do not result from differences in sensory stimulation interfering with motor performance, but are most likely of neural origin. Furthermore, it ensured that stimulation effects were comparable between subjects and groups.

Coil positions were logged into the neuronavigation software and maintained throughout the experiment for each stimulation site.

A stimulation over parieto‐occipital vertex with the same intensity served as control (sham) to account for unspecific stimulation effects like tactile and auditory sensation (Herwig, Cardenas‐Morales, Connemann, Kammer, & Schönfeldt‐Lecuona, 2010). Importantly, to reduce possible cortical stimulation effects in the control condition, the coil was angled at 45°, touching the skull not with the center but with the rim opposite to the handle. In this position, the coil to cortex distance is larger such that the electromagnetic field, if at all reaching the cortex, is substantially weaker and far outside the target range while the typical skin sensation is preserved (Herwig et al., 2010). Using this procedure, a recent study reported no difference in the perception of real and sham stimulation (Herwig et al., 2010). Likewise, no significant changes in neural activity and connectivity have been detected using this coil position in other rTMS protocols (Nettekoven et al., 2014).

#### 2.1.8 Data analysis

The kinematic data acquired during the TMS session was analyzed offline using the software 3DAWin (Version 1.2, MedCom, Munich, Germany) and the automated “segment analysis” tool of the 3DAWin software. The researcher performing the analysis was blinded about the stimulated site. First, recording artifacts were identified by visual inspection of the time series and discarded from further analysis. Then, kinematic data were filtered with standard filter bandwidths (for further technical details see (Nowak et al., 2007)), analyzed on a single trial level, and then averaged across trials.

#### 2.1.9 Pointing task

For the pointing task, we analyzed the pointing frequency based on the movements in the sagittal (*y*‐) direction of marker I. Of note, the length of the pathways (mm) covered by the subject were analyzed in *x*‐, *y*‐, and *z*‐directions and calculated as 3D Euclidean distances. Furthermore, we calculated the deviation from the target by subtracting the actual covered distance from the ideal one (which was 160 mm). We also considered the number of inversion of velocity (NIV), which describes the smoothness and rhythmicity of a repetitive movement (Amengual et al., 2013), along with all three dimensions.

#### 2.1.10 Index finger‐ and hand tapping task

For the tapping conditions, motor performance was quantified by (a) tapping frequency (Hz), (b) vertical movement amplitude (mm), and (c) the number of inversion of velocity (NIV). The data used for all analyses were obtained from the *z*‐axis of marker I which was attached to the index finger.

#### 2.1.11 Statistical analysis

Statistical analyses were performed using the software package SPSS (Statistical Package for the Social Science, version 23, IBM). To assess age‐related differences between the two groups for the resting motor threshold and motor performance at baseline, we computed independent two‐sided *t* test. Of note, to isolate region‐specific stimulation effects, we initially calculated the ratio to the difference between verum and sham relative to sham ([VERUM‐SHAM]/SHAM), which were then used for all further analyses. Stimulation effects for each task were evaluated using repeated measures analyses of variance (rm‐ANOVA) including the within‐subject factor STIMULATION SITE (three levels: M1, dPMC, IPS) and the between‐subject factor GROUP (two levels: young and old). Post hoc *t* tests were used to elucidate significant effects (*p* < .05). Furthermore, correlation analyses were computed to reveal relationships between significant TMS effects and baseline motor performance. Of note, due to our explicit interests on age‐specific differences, we also computed linear correlations for both age groups separately.

All *t* tests were Bonferroni‐corrected for multiple comparisons. In addition, we reported the effect sizes.

### 2.2 Post hoc control experiment

After completing and analyzing all data of the main experiment, we decided to add a control experiment to validate and extend our conclusions drawn from the main experiment concerning the age‐related shift identified for ipsilateral primary motor cortex and dorsal premotor cortex. We were able to reassess a subgroup of subjects which also participated in the main experiment (*n* = 13; five young healthy subjects (three females), mean age 29.4 ± 4.3 *SD* years; range: 25–34 years and eight older healthy subjects (one female), mean age 63.1 ± 5.7 *SD* years; range: 56–70 years) for a follow‐up experiment aiming at measuring interhemispheric inhibition (IHI) exerted by ipsilateral M1 and dPMC on contralateral M1.

To assess IHI, we employed a paired‐pulse stimulation technique using two Magstim 200 machines (Magstim Co., Ltd, Whitland, United Kingdom), each equipped with a 70 mm figure‐of‐eight alpha coil (Ferbert et al., 1992). The test stimulus (TS) was delivered over the left primary motor cortex. The conditioning stimulus (CS) was delivered to right, respectively ipsilateral, primary motor cortex or right dorsal premotor cortex. The interstimulus interval between the conditioning and the test stimulus was 10 ms (Ni et al., 2009; Rossini et al., 2015), which is assumed to probe direct transcallosal connections (Chen, 2004; Chen, Yung, & Li, 2003). The coordinates of the ipsilateral TMS targets (M1, dPMC) were identical to those used in the main experiment. The contralateral M1 coordinate was equivalent to the left motor hotspot as defined above. For dPMC‐M1 stimulation, both coils were orientated as described in the main experiment for the corresponding stimulation sites. By contrast, for M1‐M1 stimulation the coils were orientated in a latero‐medial direction inducing a medially directed current to avoid spatial overlapping of the two coils. Coil positions were logged into the neuronavigation software and maintained throughout the experiment for each stimulation site.

Stimulation intensities were determined separately for each hemisphere and orientation. The test stimuli were applied at the minimum intensity required to evoke a MEP of 1 mV peak‐to‐peak amplitude. The conditioning stimuli were applied at 100% RMT (see above for details on defining the RMT). Although M1‐M1 interhemispheric inhibition is usually assessed with a suprathreshold CS, we decided for this CS‐intensity because we primarily aimed at comparing M1‐M1‐IHI and dPMC‐M1‐IHI, and therefore sought to have comparable stimulation parameters with similar tactile and acoustic effects between stimulation sites (Bäumer et al., 2009; Bestmann et al., 2010; Mochizuki, Huang, & Rothwell, 2004). Importantly, our pre‐experimental tests confirmed that a 100% RMT CS and a latero‐medial coil orientation is sufficient to evoke significant M1‐M1‐IHI.

For the experiment, 15 trials per stimulation site were recorded in an interleaved design. MEPs were recorded using surface EMG from the right FDI as described for the main experiment. IHI was calculated on an individual trial basis for each of the stimulation conditions (dPMC‐M1; M1‐M1) separately, that is, by computing the ratio between the mean peak‐to‐peak amplitude of the conditioned and unconditioned MEPs (Ferbert et al., 1992; Mochizuki et al., 2004). We first eliminated outliers which were attributable to erroneous trials when the EMG signal showed some level of muscle preactivity. Subsequently, we excluded all trials of unconditioned MEPs with EMG response lower than 50 μV, and the corresponding conditioned MEP, from our analyses, since these trials cannot be considered as valid test stimuli. Together, these steps removed 4.3% of the data. Analyses revealed that the data of conditioned and unconditioned MEPs for both stimulation sites were normally distributed (*p* > .1, Shapiro Wilk test). Therefore, IHI values for each stimulation site were compared using repeated measures analyses of variance (rm‐ANOVA) including the within‐subject factor STIMULATION SITE (two levels: M1, dPMC) and the between‐subject factor GROUP (two levels: young, old). Post hoc two‐sided *t* tests were used to reveal significant between‐group effects (*p* < .05). In addition, Pearson correlations were computed to elucidate linear relationships between interhemispheric inhibition and significant TMS effects of ipsilateral M1 and dPMC.

## 3 RESULTS

### 3.1 Cortical excitability

Resting motor threshold did not significantly differ between the young (53.1% MSO ± 11.9% *SD*) and the older subjects (48.7% MSO ± 12.4% *SD*) (*p* = .28, *d* = 0.38, *t*_(30)_ = 1.09).

### 3.2 Age difference of motor performance at baseline

**Figure 4:**
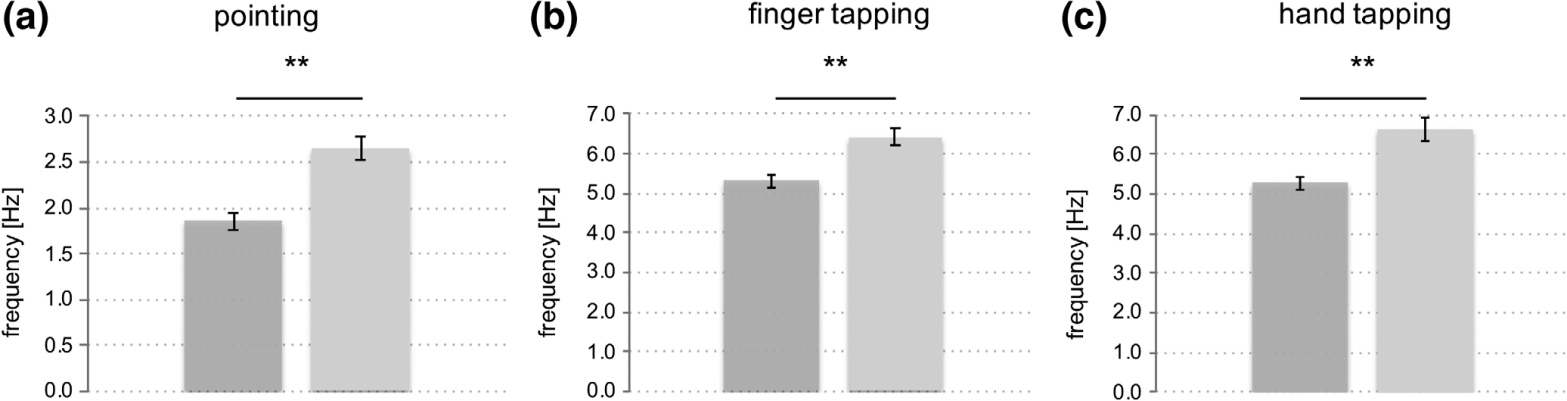
Between‐group differences in motor performance in the sham condition. (a) Pointing frequency, (b) maximum finger tapping frequency, (c) maximum hand tapping frequency, under the control condition (sham stimulation). Importantly, there were no significant differences concerning task accuracy as assessed by NIV (not shown due to no significant effect); (**p ≤ .001, two‐sided *t* test; error bars: standard error of the mean)

We first compared behavioral data between “young” and “old” in the control condition (sham) as an index of motor performance in the absence of a specific neural perturbation (Nettekoven et al., 2014). As expected, for the pointing task, we found a robust between‐group difference concerning the pointing frequency. Older subjects showed a significant slowing compared to younger subjects (*p* < .001, *d* = 0.86, *t*_(24.42)_ = 4.79) (Figure 4). Moreover, a between‐group difference for pointing NIV—a parameter for smoothness and rhythmicity of a movement—was evident with reduced movement smoothness in the older subjects (*p* = .02, *d* = 0.65, *t*_(18.14)_ = 2.99). However, we found no between‐group difference for pointing accuracy, as assessed by absolute deviation from the target (*p* = 1.0, *d* = 0.21, *t*_(30)_ = 0.61).

For both tapping conditions (i.e., finger tapping and hand tapping) we found a between‐group difference with respect to tapping frequency, with a significantly slower tapping performance in older subjects compared to young subjects (finger tapping: frequency *p* = .002, *d* = 0.67, *t*_(30)_ = 3.71; hand tapping: frequency *p* < .001, *d* = 0.71, *t*_(21.95)_ = 4.02) (Figure 4). By contrast, tapping smoothness and rhythmicity as assessed by tapping NIV did not significantly differ between groups (finger tapping: *p* = .22, *d* = 0.61, *t*_(16.7)_ = 1.77; hand tapping: *p* = .40, *d* = 0.48, *t*_(30)_ = 1.31). In summary, we found a task‐independent deterioration of movement frequency as well as a reduced rhythmicity during the pointing task in the group of old subjects, consistent with the expected decline in motor performance with higher age.

To rule out that fatigue or learning effects impacted upon motor performance, we compared means of motor performance of the first block to the ones of the last block and found no significant difference neither for young nor for older subjects (young subjects: pointing accuracy: *p* = .30, *d* = 0.16, *t*_(14)_ = 1.09; finger tapping frequency: *p* = .45, *d* = 0.07, *t*_(14)_ = 0.78, hand tapping frequency: *p* = .47, *d* = 0.06, *t*_(14)_ = 1.57; older subjects: pointing accuracy: *p* = .90, *d* = 0.02, *t*_(16)_ = 0.13; finger tapping frequency: *p* = .52, *d* = 0.11, *t*_(16)_ = 0.63; hand tapping frequency: *p* = .79, *d* = 0.04, *t*_(16)_ = 0.43). This also indicates that TMS intervention effects were stable across the entire experiment, with no evidence for after‐effects or additive effects with higher number of administered TMS pulses.

### 3.3 TMS effects on motor performance during the pointing task

The analysis of the pointing task did not reveal any significant main or interaction effects for pointing frequency (repeated measures ANOVA: main effect STIMULATION SITE: *F*_(2,60)_ = 0.33, *p* = .72, *η* ^2^ = 0.01; interaction effect STIMULATION SITE × GROUP: *F*_(2,60)_ = 0.52, *p* = .6, *η* ^2^ = 0.02; main effect GROUP: *F*_(1,30)_ = 3.34, *p* = .08, *η* ^2^ = 0.1). Hence, younger and older subjects showed no region‐specific task‐effect.

**Figure 5:**
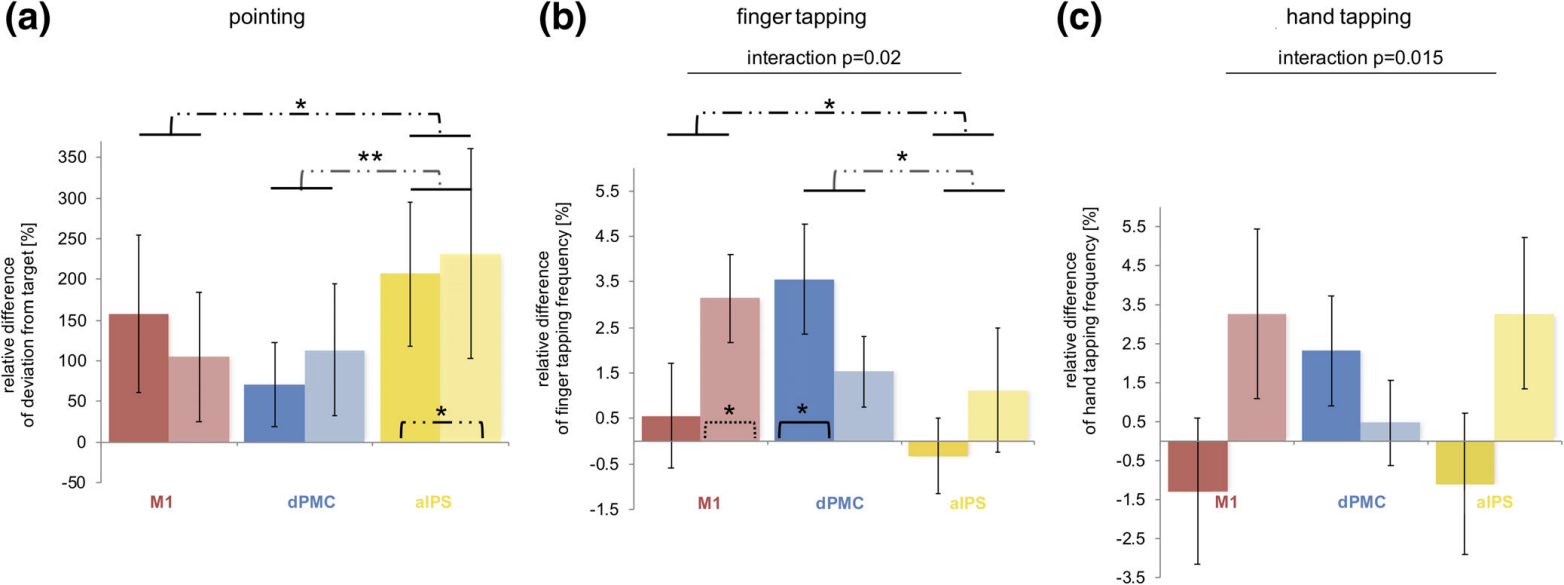
TMS effects on motor performance at different stimulation sites. Dissociation with regard to age and stimulation site for (b) finger tapping frequency: Young subjects (brighter columns and dash lines) only improved their finger tapping frequency upon interference with M1 (*p* = .018, one‐sample two‐sided *t* test), while older subjects (darker columns and solid lines) exclusively enhanced it during dPMC‐stimulation (*p* = .027, one‐sample two‐sided *t* test) (ANOVA: main effect [STIMULATION SITE] *p* = .039, interaction effect [STIMULATION SITE × GROUP] *p* = .02, dependent *t* test: M1xIPS: *p* = .04; dPMCxM1: *p* = .39; dPMCxIPS: *p* = .03); and for (c) hand tapping frequency: Again, TMS over dPMC improved tapping frequencies of older subjects while young subjects improved hand tapping frequency upon interference with M1 and IPS (ANOVA: interaction effect [STIMULATION SITE × GROUP] *p* = .015). (a) By contrast, TMS upon IPS reduced all subjects' accuracy as the target deviation increased irrespective of age (dependent *t* test: IPSxdPMC: *p* = .01; IPSxM1: *p* = .05; M1xdPMC: *p* = .43); (**p < .01, *p ≤ .05, asterisks within columns mark results of one‐sample *t* tests, error bars: standard error of the mean)

However, for deviation from pointing target, we found a significant main effect involving the factor STIMULATION SITE (*F*_(2,60)_ = 3.59, *p* = .03, *η* ^2^ = 0.11) (interaction effect STIMULATION SITE × GROUP: *F*_(2,60)_ = 0.547, *p* = .58, *η* ^2^ = 0.02; main effect GROUP: *F*_(1,30)_ = 0.002, *p* = .97, *η* ^2^ = 0). Post hoc dependent two‐sample *t* tests revealed a significant effect for IPS‐stimulation when comparing the three stimulation sites (IPS vs. dPMC: *p* = .01, *d* = 0.36, *t*_(31)_ = −2.97; IPS vs. M1: *p* = .05, *d* = 0.22, *t*_(31)_ = 1.80; M1 vs. dPMC: *p* = .43, *d* = 0.14, *t*_(31)_ = 0.79), indicating that subjects showed a greater deviation from the given target upon TMS interference with ipsilateral IPS. Importantly, this effect also significantly differed from the control condition (IPS: *p* = .027, *d* = 0.51, *t*_(31)_ = 2.90; M1: *p* = .12, *d* = 0.38, *t*_(31)_ = 2.13; dPMC: *p* = .17, *d* = 0.35, *t*_(31)_ = 1.98) (Figure 5).

In addition, a similar effect was evident with regard to NIV of the pointing task. We found a significant main effect for STIMULATION SITE (*F*_(2,60)_ = 4.64, *p* = .01, *η* ^2^ = 0.13) but no main effect for GROUP (*F*_(1,30)_ = 2.04, *p* = .16, *η* ^2^ = 0.06) or interaction for STIMULATION SITE × GROUP (*F*_(2,60)_ = 0.243, *p* = .785, *η* ^2^ = 0.01). Although subjects tended to increase the NIV upon interference with IPS and dPMC compared to M1‐stimulation (IPS vs. M1: *p* = .01, *d* = 0.51, *t*_(31)_ = −2.73; M1 vs. dPMC: *p* = .04, *d* = 0.42, *t*_(31)_ = −2.11; IPS vs. dPMC: *p* = .37, *d* = 0.16, *t*_(31)_ = −0.90), only interference with ipsilateral IPS tended to differ from the control condition (ipsilateral M1: *p* = .23, *d* = 0.02, *t*_(31)_ = 1.21; ipsilateral dPMC: *p* = .21, *d* = 0.23, *t*_(31)_ = 1.29; ipsilateral IPS: *p* = .098, *d* = 0.30, *t*_(31)_ = 1.71).

### 3.4 TMS effects on motor performance during the finger tapping task

Comparing the sham‐normalized TMS data, we found a significant main effect of STIMULATION SITE (*F*_(2,60)_ = 3.43, *p* = .039, *η* ^2^ = 0.10) and a significant interaction effect for STIMULATION SITE × GROUP (*F*_(2,56)_ = 3.98, *p* = .02, *η* ^2^ = 0.12) for finger tapping frequency (main effect GROUP: *F*_(1,30)_ = 0.31, *p* = .58, *η* ^2^ = 0.1), indicating an age‐dependent TMS effect for at least one of the three stimulation sites. Post hoc *t* tests revealed a double dissociation with respect to region and age which further elucidated this interaction effect: For younger subjects, we found a significant increase in frequency upon ipsilateral M1‐interference (*p* = .018, *d* = 0.84, *t*_(14)_ = 3.25, one‐sample two‐sided *t* test) but not for interference with ipsilateral dPMC (*p* = .22, *d* = 0.50, *t*_(14)_ = 1.92) and ipsilateral IPS (*p* = .43, *d* = 0.20, *t*_(14)_ = 0.81). By contrast, older subjects showed an increase in tapping frequency during ipsilateral dPMC‐interference (*p* = .027, *d* = 0.72, *t*_(16)_ = 2.97), but did not upon TMS‐induced disturbance of ipsilateral M1 and IPS (M1: *p* = .63, *d* = 0.12, *t*_(16)_ = 0.49; IPS: *p* = .71, *d* = 0.09, *t*_(16)_ = 0.38) (Figure 5).

Importantly, we did not find TMS effects for finger tapping amplitude (main effect of STIMULATION SITE: *F*_(2,60)_ = 0.31, *p* = .74, *η* ^2^ = 0.01; interaction effect for STIMULATION SITE × GROUP: *F*_(2,60)_ = 1.01, *p* = .37, *η* ^2^ = 0.03; main effect GROUP: *F*_(1,30)_ = 0.59, *p* = .45, *η* ^2^ = 0.02) or NIV of finger tapping (main effect of STIMULATION SITE: *F*_(2,60)_ = .367, *p* = .649, *η* ^2^ = 0.01; interaction effect for STIMULATION SITE × GROUP: *F*_(2,60)_ = 0.858, *p* = .429, *η* ^2^ = 0.03; main effect GROUP: *F*_(1,30)_ = 1.97, *p* = .17, *η* ^2^ = 0.06), indicating that the improvement in frequency was not accompanied by reduced tapping amplitude or movement smoothness.

Furthermore, there was no significant correlation between baseline finger tapping frequency and the TMS effect upon ipsilateral M1‐interference (all *p* > .1). Yet, we found a significant linear relationship between changes of finger tapping frequency evoked by ipsilateral dPMC‐stimulation and baseline finger tapping (*r* = −.40, *p* = .04). However, when testing both age groups separately, we found that this correlation was primarily driven by the old subjects (young group: *r* = .08, *p* = .76; old group: *r* = −.66, *p* = .008). Further correlation analyses, especially with age, did not reveal any significant relationship.

**Figure 6:**
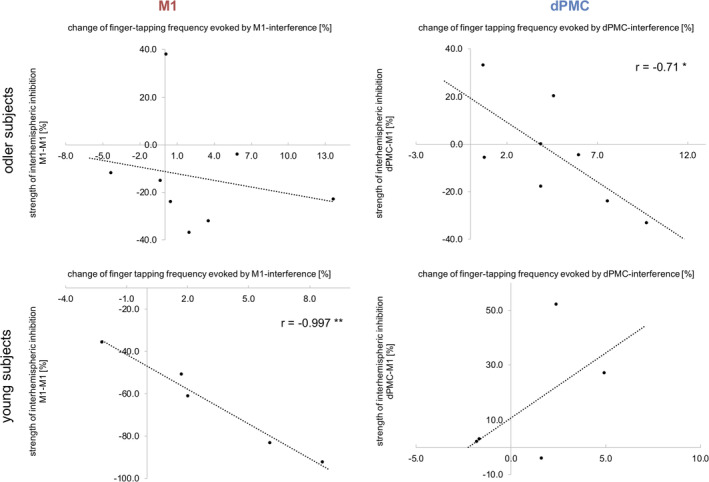
Correlation analyses between strength of IHI and TMS effect. The strength of interhemispheric inhibition between both M1 correlated negatively with the change of finger tapping frequency evoked by TMS interference with ipsilateral M1 only in young healthy individuals. By contrast, in older subjects IHI between ipsilateral dPMC and contralateral M1 correlated negatively with the effect of ipsilateral dPMC‐interference for finger tapping frequency; (*p < .05)

**Figure 7:**
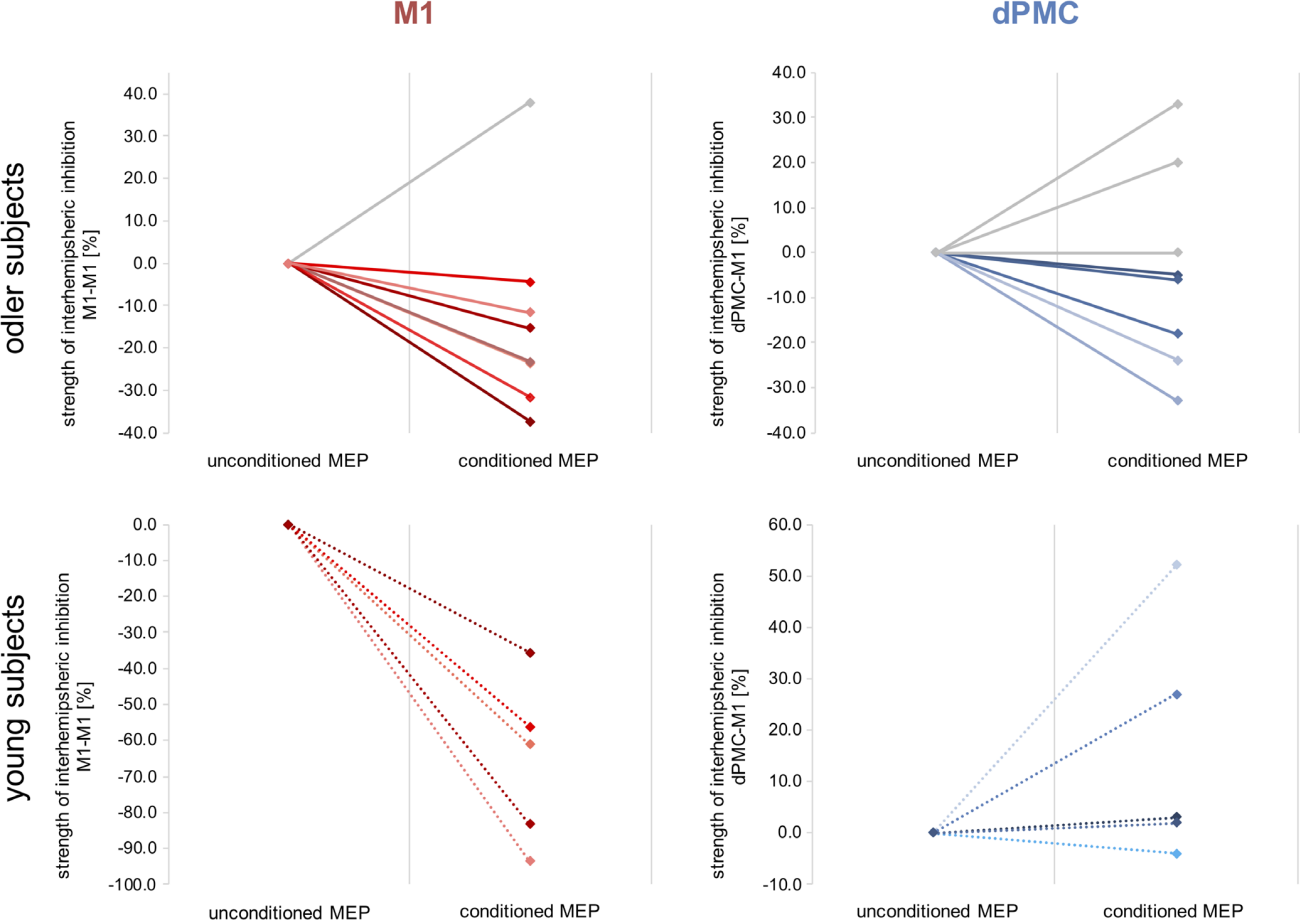
Individual data of inhibition effects. The single subject data of interhemispheric inhibition between M1‐M1 and dPMC‐M1. Please note, that older subjects featuring interhemispheric inhibitory influences are marked with color

Subsequently, we sought to link our findings for the finger tapping task to interhemispheric inhibition effects. Repeated measures ANOVA indicated a significant main effect for STIMULATION SITE (*F*_(1,11)_ = 19.94, *p* = .001, *η* ^2^ = 0.64) and a significant interaction effect for STIMULATION SITE × GROUP (*F*_(1,11)_ = 12.41, *p* = .005, *η* ^2^ = 0.53) (main effect GROUP: *F*_(1,11)_ = 4.01, *p* = .07, *η* ^2^ = 0.27), thereby pointing to an age‐dependent difference in interhemispheric inhibition. A post hoc *t* test revealed a double dissociation for region and age, hence resembling TMS effects observed for finger tapping frequency: For interhemispheric inhibition from ipsilateral M1 to contralateral M1, we found a significant group difference between young and old subjects (*p* = .002, *d* = 2.27, *t*_(10)_ = 3.95). While young participants showed strong M1‐M1 IHI with significantly reduced conditioned MEPs (33.98% ± 22.87% *SD*; *p* = .016, *d* = 1.62, *t*_(4)_ = 4.01, two‐sample *t* test between unconditioned MEPs and conditioned MEP), IHI in older subjects was diminished compared to young individuals (86.31% ± 23.44% *SD*; *p* = .31, *d* = 0.25, *t*_(7)_ = 1.10). Consistent with this result, we found a significant linear relationship between individual changes of finger tapping frequency evoked by ipsilateral M1‐stimulation and interhemispheric M1‐M1 inhibition only in young subjects (young group: *r* = −.997, *p* = .002; old group: *r* = −.22, *p* = .60) (Figure 6). Although, for interhemispheric inhibition from ipsilateral dPMC to contralateral M1, we found a nonsignificant between‐group difference (*p* = .14, *d* = 0.54, *t*_(11)_ = 1.57), young subjects featured on average facilitatory dPMC‐influences in (116.1% ± 23.4% *SD*), whereas older participants showed a decrease of facilitation compared to young subjects with a trend toward inhibition (94.0% ± 22.0% *SD*). Plotting the individual data revealed inhibitory influences in a relevant number of older subjects while none of the young subjects showed inhibitory dPMC‐M1 interactions (Figure 7). Moreover, we found a significant correlation between the TMS effects during ipsilateral dPMC‐stimulation in older but not in younger participants (young group: *r* = .30, *p* = .59; old group: *r* = −.71, *p* = .049), indicating that in particular older individuals featuring stronger interhemispheric inhibition were more susceptible to interference with ipsilateral dPMC (Figure 6). Further correlation analyses between age and IHI did not reveal any significant relationship. Hence, the IHI analyses support the findings for finger tapping performance with a differential effect of age on the contribution of ipsilateral M1 and dPMC.

### 3.5 TMS effects on motor performance during the hand tapping task

For hand tapping frequency, no significant main effect was evident for the factor STIMULATION SITE (*F*_(2,60)_ = 0.06, *p* = .94, *η* ^2^ = 0.002). However, paralleling the findings of finger tapping, we found an interaction effect for STIMULATION SITE × GROUP (*F*_(2,60)_ = 4.55, *p* = .015, *η* ^2^ = 0.13) (main effect GROUP: *F*_(1,30)_ = 1.32, *p* = .26, *η* ^2^ = 0.04). Likewise, hand tapping frequencies tended to be faster in younger subjects when interfering with ipsilateral M1 and, additionally, with ipsilateral IPS, while older subjects showed an increase in tapping frequency under ipsilateral dPMC‐stimulation. However, effects were much weaker as indicated by the nonsignificant post hoc one‐sample *t* tests (young group: ipsilateral M1: *p* = .16, *d* = 0.39, *t*_(14)_ = 1.50; ipsilateral dPMC: *p* = .68, *d* = 0.11, *t*_(14)_ = 0.43, ipsilateral IPS: *p* = .11, *d* = 0.44, *t*_(14)_ = 1.69) (old group: ipsilateral M1: *p* = .50, *d* = 0.17, *t*_(16)_ = 0.69; ipsilateral dPMC: *p* = .12, *d* = 0.40, *t*_(16)_ = 1.63; ipsilateral IPS: *p* = .55, *d* = 0.15, *t*_(16)_ = 0.61) (Figure 5). Likewise, neither M1‐M1 nor dPMC‐M1 IHI correlated with hand tapping effects.

## 4 DISCUSSION

We here applied online TMS interference to investigate the functional relevance of ipsilateral frontoparietal regions for motor performance in young and older subjects. At the behavioral level, older subjects featured a significant slowing in all investigated motor tasks. Importantly, there was no group difference concerning movement accuracy, indicating that preserved movement accuracy was presumably achieved via reduced movement speed. Interfering with neural activity during task performance yielded differential effects depending on age and stimulation site. A novel finding of our study is the differential relevance of ipsilateral M1 and ipsilateral dPMC on fast repetitive movements depending on age, which was paralleled by age‐related changes of IHI. In particular, the current findings are compatible with an age‐related functional shift from ipsilateral M1 toward ipsilateral dPMC in the attempt to maintain tapping performance at high frequencies in older subjects, while interfering with anterior superior parietal cortex activity affected visuomotor performance independent of age.

### 4.1 HAROLD and the motor system: The role of ipsilateral primary motor cortex in age

In young adults, there is compelling evidence that ipsilateral M1 contributes to motor tasks of increasing complexity. Neuroimaging‐ as well as TMS studies have revealed that tasks particularly requiring fine‐tuned temporal regulation or high muscle selectivity involve ipsilateral M1 (Chen, Gerloff, Hallett, & Cohen, 1997; Davare, Andres, et al., 2006; Hummel et al., 2003; Kim et al., 1993; Verstynen, 2004). In agreement with these findings, we here observed that interference with ipsilateral M1 significantly impacted upon both finger and hand tapping tasks in young healthy subjects. Specifically, ipsilateral M1‐disruption led to higher tapping frequencies without changes in tapping amplitudes or movement smoothness in both tasks, suggesting an actual improvement of motor performance. Since the majority of previous online TMS studies have reported detrimental behavioral responses for interfering with ipsilateral M1 (Chen et al., 1997; Davare, Andres, et al., 2006; Foltys et al., 2001), our present data—at least at first sight—seems to be at odds with these previous studies. However, the fact that we observed an increase of frequency upon ipsilateral M1 interference not only for the finger tapping but also for the hand tapping task, which served as an additional internal control task, strengthen our data. Moreover, Davare and colleagues (Davare, Duque, Vandermeeren, Thonnard, & Olivier, 2006) have shown that disruption of ipsilateral M1 could either advance or delay muscle recruitment dependent on the timing of TMS interference, adding additional support for differential effects of online TMS interference.

From a mechanistic perspective, online TMS applied to ipsilateral M1 might have modulated interhemispheric inhibition between bilateral M1 which is in accordance with the significant relationship that we found between TMS effects of ipsilateral M1 stimulation and interhemispheric inhibition exerted by ipsilateral M1 in young subjects. In addition, TMS studies as well as connectivity analyses based on task fMRI data have also provided converging evidence that each M1 exerts reciprocal influences onto its contralateral homolog, which seems to be crucial for motor control and muscle recruitment (Di Lazzaro et al., 1999; Ferbert et al., 1992; Meyer et al., 1995).

Accordingly, the interhemispheric influences exerted from ipsilateral M1 onto the contralateral M1 have been shown to be initially inhibitory at rest, to decrease progressively when approaching muscle contraction, and to reverse to facilitation before and during muscle contraction (Davare, Andres, et al., 2006; Duque et al., 2005; Murase, Duque, Mazzocchio, & Cohen, 2004). Therefore, depending on the timing of TMS disruption relative to movement execution, it could either advance or delay muscle recruitment (Davare, Duque, et al., 2006). Accordingly, with respect to the present study, TMS interference seems to have affected the inhibitory role of M1 leading to a more effective disinhibition of contralateral M1, thereby increasing tapping frequency in young subjects (Davare, Andres, et al., 2006; Volz et al., 2017).

For older subjects, neuroimaging studies have typically revealed increased activity in ipsilateral M1 compared to young individuals during hand‐motor tasks (Mattay et al., 2002; Riecker et al., 2006; Ward & Frackowiak, 2003), consistent with an extension of the *HAROLD* model into the motor domain. We could show that interhemispheric inhibition between both M1 is attenuated in older subjects compared to young individuals, which is supported by a number of studies (Coppi et al., 2014; Talelli, Ewas, Waddingham, Rothwell, & Ward, 2008; Talelli, Waddingham, Ewas, Rothwell, & Ward, 2008). Hence, less hemispheric asymmetry might be a consequence of reduced interhemispheric inhibition effects leading to disinhibition of neural activity in ipsilateral M1 (Langan et al., 2010; Talelli, Greenwood, & Rothwell, 2006; Ward et al., 2008). As several authors could not link over‐activation of ipsilateral M1 to better motor performance, higher activity of this area might also represent unspecific disinhibition rather than compensatory mechanisms (Riecker et al., 2006; Talelli, Ewas, et al., 2008).

Taken together, these findings are relevant for the interpretation of our data, as unlike in younger subjects, we could not find a significant functional involvement of ipsilateral M1 in the employed motor tasks in older subjects.

### 4.2 PASA and the motor system: Premotor shift in aging

*PASA* would predict an aging‐associated shift from posterior to anterior regions, not only in the contralateral but also ipsilateral hemisphere (Davis et al., 2008; Michely et al., 2018), which is paralleled by a key finding of our study—an age‐related anterior shift within the motor network. The disruption of ipsilateral dPMC‐activity affected motor performance in older but not younger subjects, indicating a shift from ipsilateral M1 to premotor cortex during repetitive high‐frequency movements.

One might argue that the observed stimulation effects in dPMC in the older group may result from stimulating adjacent M1 neurons, or unspecific facilitation effects due to the sensory input associated with TMS (Duecker & Sack, 2013). However, since we observed a double dissociation between groups and brain areas, this hypothesis appears rather unlikely.

Given that neuroimaging studies have frequently demonstrated a greater activation of ipsilateral premotor regions during motor tasks with advancing age (Heuninckx, Wenderoth, & Swinnen, 2008; Mattay et al., 2002; Riecker et al., 2006; Ward & Frackowiak, 2003) and we here found an improvement of motor performance, one interpretation of the present data is an detrimental influence of ipsilateral premotor cortex activity upon contralateral M1, which is released by TMS interference—similar to what has been described for neural over‐activity in the contralesional hemisphere in stroke patients (Grefkes et al., 2008; Murase et al., 2004; Rehme, Fink, Cramon, Y, & Grefkes, 2011; Volz et al., 2017).

However, we would like to challenge this interpretation, especially since we could also detect an improvement in task performance evoked by TMS in young healthy individuals in whom it seems rather unlikely that ipsilateral areas hold a detrimental role for motor performance. As the effects evoked by the stimulation are thought to be influenced by several factors like timing of stimulation onset or stimulation intensity (Davare, Duque, et al., 2006; Foltys et al., 2001; Jahanshahi & Rothwell, 2000; Silvanto & Cattaneo, 2017; Walsh & Cowey, 2000), the TMS‐induced effects of a distinct region allow to draw conclusions about the causal and functional involvement of this region, but does not necessarily determine a beneficial or maladaptive role.

Highly similar to ipsilateral M1, also ipsilateral dPMC modulates the activity of contralateral M1 through inhibitory and facilitatory influences (Bäumer et al., 2009; Hinder, Fujiyama, & Summers, 2012; Koch et al., 2006; O'Shea, Sebastian, Boorman, Johansen‐Berg, & Rushworth, 2007). Most likely these effects are mediated on the anatomical basis of direct commissural fibers from dorsal premotor cortex to contralateral M1, which have been confirmed in monkeys (Boussaoud, Tanné‐Gariépy, Wannier, & Rouiller, 2005; Jenny, 1979; Marconi, Genovesio, Giannetti, Molinari, & Caminiti, 2003). This notion receives additional support since IHI with short interstimulus intervals is suggested to probe direct transcallosal pathways (Chen, 2004; Chen et al., 2003; Hinder et al., 2012).

Hinder and colleagues found that older subjects particularly rely on a stronger modulation of inhibition toward facilitation to release the motor signal to the contralateral hand (Hinder et al., 2012; Levin, Fujiyama, Boisgontier, Swinnen, & Summers, 2014). Although we were able to assess IHI only in a limited subgroup of the original cohort, we here found hints that, compared to young subjects, interhemispheric interactions between ipsilateral dPMC and contralateral M1 of older participants were shifted toward inhibition. Therefore, a similar modulation stated for ipsilateral M1 in young subjects, namely a TMS‐induced disruption of the inhibition exerted from ipsilateral dPMC onto contralateral M1, may have led to a more effective disinhibition of contralateral M1 and mediated the improvement of motor performance in older subjects. This notion receives further support from the observed relationship between TMS effects of ipsilateral dPMC stimulation and IHI exerted by ipsilateral dPMC in older subjects. Hence, during repetitive high‐frequency movements, ipsilateral dPMC presented a similar TMS profile in older subjects, analogous to the response of younger subjects in ipsilateral M1. Furthermore, the interhemispheric inhibition exerted from ipsilateral dPMC in older subjects revealed a similar relationship to the TMS effect of dPMC interference, paralleling the results of interhemispheric inhibition and TMS effect of ipsilateral M1 in young subjects.

Notably, the IHI experiment bears some limitations such as the small sample size which reduces the generalizability of our data and the fact that both experiments were not conducted on the same day. Nevertheless, the age‐related differential findings fit the results obtained in the main experiment and support our interpretation of an age‐related functional shift for finger tapping performance.

So far, neuroimaging studies have revealed that premotor cortex is more activated during motor tasks in older individuals (Heuninckx, 2005; Heuninckx et al., 2008; Ward et al., 2008) and our data is compatible with a causal involvement of dorsal premotor cortex in motor tasks in older individuals. Given that we found a relationship between baseline motor performance and changes of finger tapping frequency evoked by ipsilateral premotor cortex stimulation, dorsal premotor cortex seems to be particularly relevant in older subjects with poor motor performance. Therefore, the age‐related functional premotor shift, although potentially representing a reorganization due to structural and biochemical changes in the motor system (Seidler et al., 2015; Talelli, Waddingham, et al., 2008; Ward et al., 2008), could not be considered as fully compensatory from a behavioral perspective. Hence, compensation may only be feasible to a limited degree, and those subjects with overt reduction in motor performance, indicating insufficient compensation, are especially sensitive to TMS interference.

### 4.3 The role of the ipsilateral anterior intraparietal sulcus

For the visuomotor pointing task, we found a similar stimulation effect for TMS interference with ipsilateral IPS for both young and older individuals. There is a large body of literature from both monkeys and humans reporting that anterior intraparietal cortex is firmly engaged in visuospatial aspects of visually guided hand‐motor tasks (Binkofski et al., 1998; Binkofski et al., 1999; Culham et al., 2003; Grefkes et al., 2004; Kalaska, Cisek, & Gosselin‐Kessiby, 2003). Moreover, IPS serves as an interface for the integration of visual, motor, somatosensory, and spatial information (Grefkes & Fink, 2005). Therefore, it seems plausible, that in our study interference with IPS impaired the target accuracy of reaching movements relying on visuomotor integration. These data are compatible with previous findings reported by Davare and colleagues who showed that interfering with both contra‐ and ipsilateral IPS by means of single pulse TMS leads to errors in target‐directed movements, highlighting the functional involvement of bilateral IPS in young controls (Davare, Zénon, Desmurget, & Olivier, 2015). Our data showed comparable effects of ipsilateral IPS‐interference in younger and older subjects, suggesting a similar role in visually guided movements independent of age. Interestingly, despite the evidence that also posterior parietal areas including the IPS show higher task‐related activity in older subjects (Labyt et al., 2004; Ward & Frackowiak, 2003), we could not dissociate between young and older subjects the role of this region in maintaining pointing performance. However, an alternative explanation for the absence of an age‐dependent functional dissociation of IPS is that other areas than tested in the present study like, for example, inferior parietal cortex or more posterior parietal regions might be more relevant for maintaining visuomotor performance specifically in older subjects (Mattay et al., 2002; Ward, 2006).

## 5 CONCLUSION

In conclusion, we here provide evidence for differential functional roles of ipsilateral M1 and dPMC in age with a decline of relevance of M1 and a functional shift of importance toward dPMC for repetitive high‐frequency movements associated with aging. Our results apply existing models of aging‐associated changes of *PASA* and *HAROLD* to the motor system, but extend these accounts regarding the causal relevance of reorganization of neural activity in aging.

## Data Availability

The data that support the findings of this study and all custom‐written codes are available from the corresponding author upon reasonable request.
